# Bioactivities of Pseudocereal Fractionated Seed Proteins and Derived Peptides Relevant for Maintaining Human Well-Being

**DOI:** 10.3390/ijms22073543

**Published:** 2021-03-29

**Authors:** Jessica Capraro, Stefano De Benedetti, Giuditta Carlotta Heinzl, Alessio Scarafoni, Chiara Magni

**Affiliations:** Department of Food, Environmental and Nutritional Sciences, Università degli Studi di Milano, 20133 Milano, Italy; jessica.capraro@unimi.it (J.C.); stefano.debenedetti@unimi.it (S.D.B.); giuditta.heinzl@unimi.it (G.C.H.); chiara.magni@unimi.it (C.M.)

**Keywords:** seed storage proteins, peptides, anti-inflammatory, antioxidant, trypsin inhibitors, quinoa, amaranth, buckwheat, in vitro gastro-intestinal digestion

## Abstract

Food proteins and peptides are able to exert a variety of well-known bioactivities, some of which are related to well-being and disease prevention in humans and animals. Currently, an active trend in research focuses on chronic inflammation and oxidative stress, delineating their major pathogenetic role in age-related diseases and in some forms of cancer. The present study aims to investigate the potential effects of pseudocereal proteins and their derived peptides on chronic inflammation and oxidative stress. After purification and attribution to protein classes according to classic Osborne’s classification, the immune-modulating, antioxidant, and trypsin inhibitor activities of proteins from quinoa (*Chenopodium quinoa* Willd.), amaranth (*Amaranthus retroflexus* L.), and buckwheat (*Fagopyrum esculentum* Moench) seeds have been assessed in vitro. The peptides generated by simulated gastro-intestinal digestion of each fraction have been also investigated for the selected bioactivities. None of the proteins or peptides elicited inflammation in Caco-2 cells; furthermore, *all protein fractions* showed different degrees of protection of cells from IL-1β-induced inflammation. Immune-modulating and antioxidant activities were, in general, higher for the albumin fraction. Overall, seed proteins can express these bioactivities mainly after hydrolysis. On the contrary, higher trypsin inhibitor activity was expressed by globulins in their intact form. These findings lay the foundations for the exploitation of these pseudocereal seeds as source of anti-inflammatory molecules.

## 1. Introduction

Modern trends in dietary approaches and ethical and environmental sustainability concerns catalyze researchers’ interest in plant proteins as an alternative to animal proteins [1]. In this frame, the consumption of pseudocereals has increased significantly over the past few years, which is also due to their integration into vegan/vegetarian and gluten-free diets [2]. The protein content of their seeds varies from 9.1 to 16.7% in quinoa, 13.1 to 21.5% in amaranth, and 5.7 to 14.2% in buckwheat [3]. Their amino acid composition is generally well balanced, with higher contents of lysine, methionine, and cysteine than common cereals and legumes [2]. The distribution of the three aforementioned pseudocereal seed proteins according to their solubility reveals a closer relationship to legumes than to cereal proteins, since albumins and globulins are the most abundant protein species [4].

It has been well established that foods may contain molecules able to exert a variety of bioactivities, some of which are related to well-being and disease prevention in humans and animals [5,6]. Beyond their nutritional value, recent research identified the potential health benefits of food proteins and peptides. They can exert antioxidant, antihypertensive, antilipidemic, hypocholesterolemic, antimicrobial, and immune-modulating effects [7,8]. Among these peptides, many have been identified in legume and cereal seeds, including soybean (*Glycine max*), lupin (*Lupinus* ssp.), corn (*Zea mays*), horse gram (*Macrotyloma uniflorum*), cowpea (*Vigna unguiculata*), common bean (*Phaseolus vulgaris*), and lentil (*Lens culinaris*) [9,10,11,12,13,14]. However, there is still a general lack of information about the potential effects of pseudocereal proteins and their derived peptides on chronic inflammation and the various forms of oxidative stress, these being considered as major causes of age-related diseases and of some forms of cancer [15].

The present study aims to advance the knowledge of selected activities potentially exerted by pseudocereal proteins. The immune-modulating, antioxidant, and trypsin inhibitor activities of proteins from quinoa (*Chenopodium quinoa* Willd.), amaranth (*Amaranthus retroflexus* L.), and buckwheat (*Fagopyrum esculentum* Moench) seeds have been assessed in vitro, after purification and separation in different fractions. The three biological activities considered in this work are intimately linked to each other with regards to their implications on human health.

Indeed, inflammation plays an important role in the ability of the immune system to fend off pathogens and harmful agents. However, an unregulated inflammatory response can lead to tissue damage and the development of chronic inflammatory diseases [16]. Several food-derived compounds are able to modulate the immune response in humans [17]. For example, many compounds may mediate inflammation by altering the DNA-binding capacities of NF-κB, the major effector of immune response pathways, and other transcription factors [18]. NF-κB acts as a central inflammatory mediator by regulating a vast array of genes involved in the immune and inflammatory responses. It responds to a large variety of molecules, including cytokine IL-1β, and its activation induces the expression of inflammatory cytokines, chemokines, and adhesion molecules [16]. Hence, the control of the NF-κB pathway represents a potential strategy for preventing inflammation-associated diseases [17]. In the present work, the effects on cell inflammation of proteins and their peptides obtained by simulated gastro-intestinal digestion have been studied using cultivated intestinal Caco-2 cells, whose immune response was triggered by IL-1β.

The dampening of oxidative processes is of great importance to human well-being [19]. When free radicals are overproduced or the cellular defenses are impaired, biomolecules such as lipids, proteins, and DNA may be damaged by oxidative stress [20], ultimately leading to pathological conditions. Plant foods are rich in antioxidant molecules, especially phenolic compounds [6]. However, an increasing body of evidence suggests that proteins and peptides can also exert this protective effect [11,21,22,23,24,25,26,27,28,29]. The capacity of inhibition on the oxidation of cellular components can be exerted through multiple mechanisms of action, including free radical scavenging, metal ion chelation, and hydroperoxides and reactive oxygen species reduction [30]. In addition, the typical amphipathicity of most peptides allows them to act both in aqueous and lipidic systems [31].

Although protease inhibitors (PIs) have long been considered anti-nutritional compounds because of their negative effects on protein digestibility, several recent studies have shown that they may play important roles in the treatment or prevention of inflammation-associated diseases, such as some types of cancers [32,33], autoimmune diseases [34], coagulation diseases [35], metabolic syndrome, and obesity [36]. These studies focused mainly on PIs from leguminous plants, and information about PIs from pseudocereal seeds continues to remain limited [37,38,39,40,41]. It is known that serine proteases act as modulators of the immune system and inflammatory response by regulating cytokine and chemokine production. Aberrant functioning of serine proteases may contribute to the development of disorders derived from inflammatory cell activation that lead to immunological problems and excessive activation of inflammation [34]. Thus, the inhibition of serine proteases by PIs may play a role in the prevention of these diseases [42].

## 2. Results and Discussion

### 2.1. Purification of Pseudocereal Protein Fractions and Their In Vitro Digestion

**Figure 1:**
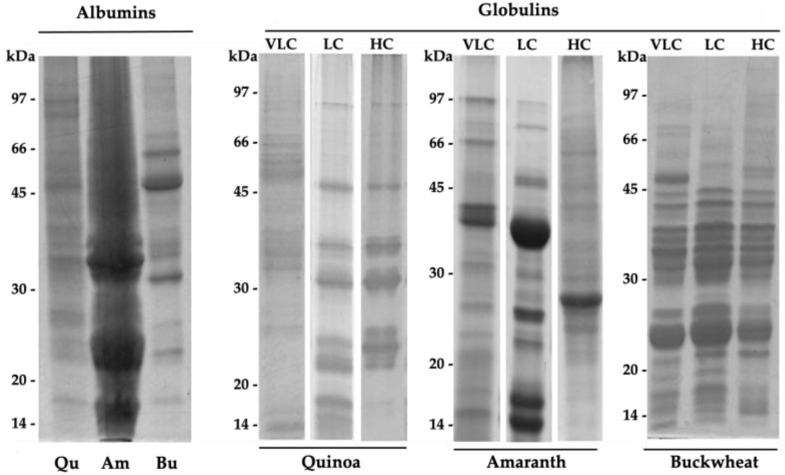
SDS-PAGE analysis of albumins and globulin fractions from quinoa (Qu), amaranth (Am), and buckwheat (Bu) seed proteins. VLC: very low charge; LC: low charge; HC: high charge.

The isolation procedure we adopted allowed us, as a first step, to obtain a water-soluble fraction, namely albumin, and a salt-soluble fraction, corresponding to globulins. Albumins and globulins are the most abundant seed proteins of pseudocereals. Amaranth, buckwheat, and quinoa contain different proportions of each [4]. Albumins include many enzymes involved in cotyledon cell metabolism and plant defense, whereas globulin proteins essentially play a storage role. Due to the low selective pressure, seed storage proteins (SSPs) show common characteristics among species. Globulins may be classified according to the sedimentation coefficient as 2S, 7–8S, and 11–13S, also known as vicilin-like and legumin-like globulin, respectively [43]. In order to visualize the distribution of the proteins in the obtained fractions, these latter were analyzed by SDS-PAGE (Figure 1). The albumin electrophoretic patterns are clearly different from those of the respective globulin fractions, confirming that the procedure allowed the differential solubilization of the two protein classes (Figure 1). 

Due to the combination of different factors, including the and the post-translational modifications, globulin polypeptide chains show size and charge heterogeneity [7]. For this reason, we further fractionated globulin by ion-exchange chromatography, according to their net surface charge at pH 8.0, in three fractions named VLC (very low charge), LC (low charge) and HC (high charge). The quinoa VLC fraction consists of a large range of different polypeptides. Notably, the SDS-PAGE pattern was very similar to that of the salt-soluble fraction obtained following isoelectric precipitation of chenopodins, as shown by Brinegar et al. [44], and very different from the two retained LC and HC fractions. These two fractions include isoforms of the 11–13S globulin chenopodin, the main quinoa SSPs [45]. The bands with molecular weights (MWs) of 35–37 and 22–25 kDa correspond to the α and β chains of chenopodin, respectively. Thus, the purification procedure we set up allowed the separation of the quinoa proteins using only a single chromatographic step.

Buckwheat VLC, LC, and HC globulin separation produced very similar patterns, with the only difference being one extra polypeptide of about 50 kDa in the VLC fraction. Again, this result is due to the charge heterogeneity of constituent polypeptides of buckwheat globulins [46,47]. The two visible main groups of polypeptides with MWs of 30–47 and 23–25 kDa likely correspond to the α and β chains of 13S globulin, the most abundant buckwheat globulin [4].

Amaranth separated globulins showed more complex polypeptide profiles than either the quinoa or the buckwheat samples. The LC fraction included bands likely attributable to 11S amaranthin. Amaranthin subunits consists of acidic (34–36 kDa) and basic (22–24 kDa) polypeptides. A polypeptide of about 55 kDa is also present in this fraction. In all probability, it is the uncleaved precursor (52–59 kDa) [48]. Vicilin-like 7S globulins are represented by several polypeptides with MWs between 15 and 90 kDa, also present in amaranth seeds [4]. Due to their more acidic pI, in our case, they are mainly distributed in the VLC fraction. A typical band of about 38 kDa is visible [49]. 

**Figure 2:**
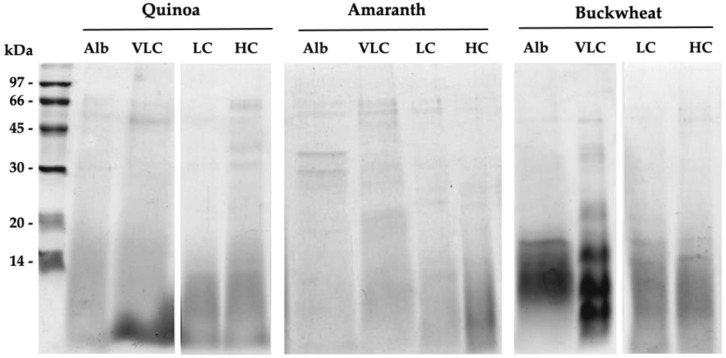
SDS-PAGE analysis of albumins and globulin fractions after simulated gastro-intestinal digestion. Alb: albumin fraction; VLC: very low-charge globulins; LC: low-charge globulins; HC: high-charge globulins.

Bioactive cryptic peptides are specific protein fragments inactive in the whole intact protein but active when released by proteolysis. Dietary proteins may supply active peptides able to promote human health and well-being [7]. The most studied cryptic peptides are those released from milk, chicken eggs, and meat protein digestion, whereas plant proteins are much less studied as source of food-derived active peptides. To obtain peptides as similar as possible to those that would be obtained following ingestion, a simulated gastro-intestinal digestion was performed using well-established procedures [50]. The proteolytic treatment caused the breakdown of proteins into fragments with molecular weights equal to or smaller than 14 kDa, as revealed by SDS-PAGE (Figure 2). In all samples, only a small number of bands with MWs higher than 30 kDa remained faintly visible.

### 2.2. The Immune-Modulating Capacity of Protein Fractions and Peptides in Caco-2 Cells 

**Figure 3:**
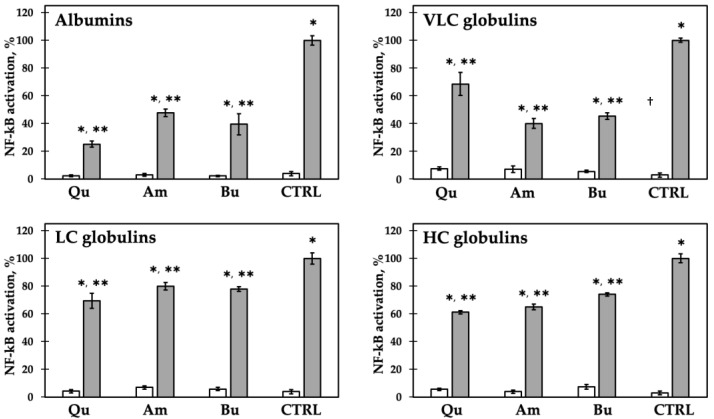
Inflammatory response of Caco-2 cells incubated with 1.0 mg/mL of each protein class without (white bars) or with (gray bars) IL-1β. Response to IL-1β alone was set as 100% (CTRL). Qu: quinoa; Am: amaranth; Bu: buckwheat. * *p* < 0.05 vs. CTRL without IL-1β; ** *p* < 0.05 vs. CTRL with IL-1β. Each experiment was performed in triplicate.

To study the immune-modulating activity of intact protein fractions, we evaluated NF-κB activation by transiently transfecting epithelial intestinal Caco-2 cells with an NF-κB luciferase reporter construct that includes five NF-κB binding sites. The presence of NF-κB-activating molecules stimulated the expression of the luciferase gene. The results are shown in Figure 3. After incubation with the protein fractions, NF-κB activation was comparable with cells incubated with the culturing media to suggest that amaranth, quinoa, and buckwheat proteins did not exert pro-inflammatory effects by themselves. Under stimulation with the pro-inflammatory cytokine IL-1β, the presence of pseudocereal protein fractions induced the decrease of inflammatory response in all cases but to different extents.

Overall, albumin and VLC globulin showed higher anti-inflammatory activity (a drop of inflammation from 31 to 74%) than LC and HC globulin fractions (from 20 to 39%). Quinoa albumin was the most active fraction, showing a 74% inflammation reduction. The immune-modulating effects of quinoa LC and HC globulin fractions are similar to those previously described [45] and are slightly higher than those of amaranth and buckwheat globulins. In the present study, we demonstrated that amaranth, buckwheat, and quinoa seed proteins potentially modulate an inflammation response by decreasing NF-κB activation. To date, very few plant proteins in the intact form are known to possess anti-inflammatory properties [45,51,52,53]. The most studied protein presently is lunasin, a 43-amino-acid peptide isolated from soybean which acts on macrophage cells by inhibiting the NF-κB pathway [54,55,56]. Recently, lunasin was detected in quinoa seeds as well and its bioactive effects were confirmed [55]. Since lunasin extraction efficiency in water is high [57], it is likely that, in our case, it is mainly present in the albumin fraction. This supports the observed high protective effect of this fraction (Figure 3). The binding of lunasin with αVβ3 integrin through an Arg-Gly-Asp (RGD) motif has been associated with inhibition of the NF-κB pathways [54]. Interestingly, the RGD/RGE motif was found in three sequences of chenopodin [45]. Searching protein sequence databases, an RGE motif was found only in sequence ABO93593.1 of buckwheat BW10KD allergen protein, corresponding to 2S albumin [58].

**Figure 4:**
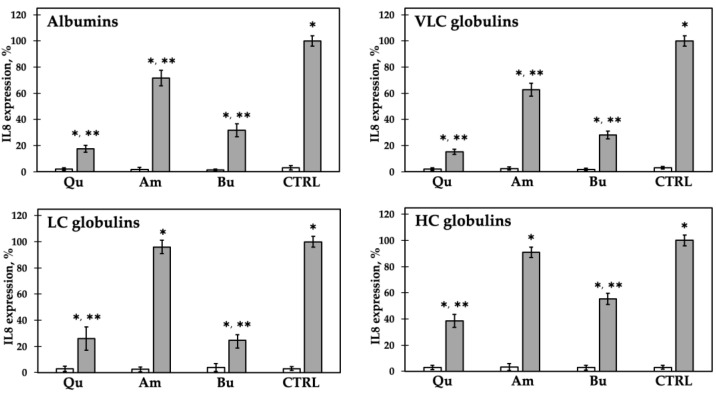
Inflammatory response of Caco-2 cells incubated with 1.0 mg/mL of peptides originated by in vitro simulated gastro-intestinal digestion of each protein class, without (white bars) or with (gray bars) IL-1β. Response to IL-1β alone was set as 100% (CTRL). Qu: quinoa; Am: amaranth; Bu: buckwheat. * *p* < 0.05 vs. CTRL without IL-1β; ** *p* < 0.05 vs. CTRL with IL-1β. Each experiment was performed in triplicate.

Finally, we assessed the potential anti-inflammatory capacity of the pool of peptides originated from the single fractionated proteins by simulated gastro-intestinal digestion. In a previous work [45], we showed that the expression of chemokine IL-8 mirrors the expression of NF-κB in undifferentiated Caco-2 cells stimulated with IL-1β and treated with quinoa chenopodin isoforms. Since the levels of NF-κB and IL-8 can be considered to be modulated in the same way, we decided to directly quantify the expression of IL-8 by qRT-PCR to evaluate the anti-inflammatory effect of the peptides (Figure 4). 

Again, the peptides alone, originating from the different samples, exerted no inflammatory activity in Caco-2 cells. Peptides from quinoa and buckwheat protein fractions were effective in lowering the inflammatory stimulus due to the presence of IL-1β with a higher extent if compared to the effect exerted by the undigested proteins. On the contrary, amaranth peptides showed a minor protective shielding. LC globulin peptides from amaranth appeared absolutely ineffective. 

### 2.3. The In Vitro Antioxidant Activity of Protein Fractions and Peptides

The antioxidant activity of peptides obtained through generic enzymatic treatments of total protein extracts from amaranth, buckwheat, and quinoa has been reported [29,59,60,61,62,63,64]. This finding, although interesting, does not provide information on the most active protein classes, on their functional implications, or on what might actually happen when proteins are ingested. Therefore, in order to obtain indications on the specifics of each fraction, we focused our attention on the radical scavenging capacity of every single pseudocereal protein fraction and of the peptides generated by their simulated gastro-intestinal digestion.

**Figure 5:**
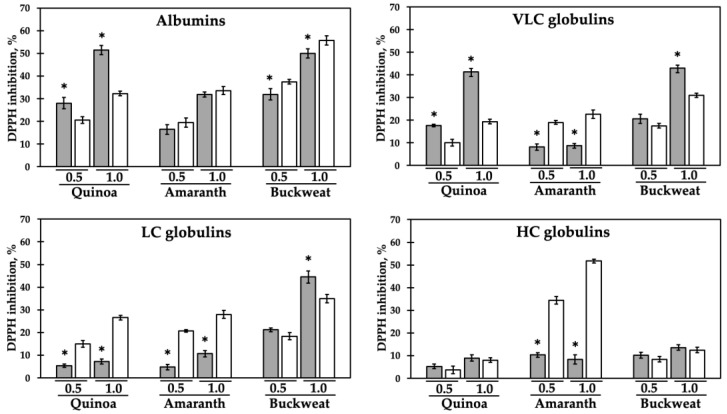
DPPH (2,2-diphenyl-1-picrylhydrazyl) radical inhibition percentage at two different protein concentrations (0.5 mg/mL and 1.0 mg/mL) of each separated protein class, in the native form (gray bars) and after simulated gastro-intestinal digestion (white bars). * *p* < 0.05 vs. digested form. Each experiment was performed in triplicate.

Figure 5 shows the DPPH radical inhibition percentage at two different protein concentrations (0.5 mg/mL and 1 mg/mL). In general, the different globulin fractions showed low antioxidant activity when tested in the undigested form (amaranth VLC, quinoa and amaranth LC, and all HC fractions). 

As described in Materials and Methods, the radical scavenging activity was measured via use of the DPPH (1,1-diphenyl-2-picrylhydrazyl) assay, a well-established method to evaluate the antioxidant potential of a compound [65]. Many in vitro methods for the assessment of antioxidant activity in foods of plant origin are available and currently used in a number of studies [66]. Although the principle of each group of antioxidant assay methods are similar, their applicability depends on various factors, including the presence of lipophilic compounds [66]. Gallego et al. [67] reported that the antioxidant activity of peptides is related more to their amino acid composition, structure, and hydrophobicity rather than their size. Indeed, studies indicate that a high radical scavenging activity of protein hydrolysates or peptides is usually associated with a high hydrophobic, aromatic, or sulphur amino acid content [28,63,68]. For all these reasons, the DPPH method was used in this study. Different protein fractions, especially from buckwheat and quinoa, showed antioxidant activity when tested in the intact form (all albumin, buckwheat VLC and LC, and quinoa VLC fractions). 

The results indicate that the effect of proteolysis on the antioxidant activity varies according to the protein species. Digested albumins from amaranth and buckwheat possess scavenging activity similar to, or slightly higher than, the undigested forms. Globulin fragmented forms showed increased activity in all the cases for amaranth and in quinoa LC, whereas in the case of buckwheat, the digested globulins scavenging potential was similar or slightly less when compared to the undigested form. Thus, digestion of these protein fractions potentially leads to the release of encrypted antioxidant peptides. Indeed, the most striking result was the observed substantial increase of antioxidant potential in all the digested globulin fractions from amaranth. An increase in the scavenging capacity as a result of the degree of proteolysis was reported for amaranth, buckwheat, quinoa, and chickpea total protein hydrolysates [25,29,59,60,61,63]. Our results allowed us to quantify the antioxidant activity of each separated protein class and to identify the protein and derived peptides fractions showing the highest activities. For example, our data indicate that the antioxidant peptides were released by LC chenopodin. These data support the findings of Vilcacundo et al. [64], who identified 17 potential antioxidant peptides from quinoa proteins, 13 of which derived from acidic and basic subunits of chenopodin.

It has been demonstrated that the fractions of hydrolyzed buckwheat proteins contained di-, tri-, and tetrameric peptides. When tryptophan, proline, valine, leucine, and phenylalanine were present, these fractions exhibited the strongest radical scavenging activity [29]. Based on these observations, we investigated the amino acid composition of albumin and globulin protein classes for each pseudocereal. Table 1 shows the sum of the percentage content of the amino acids which may be involved in redox reactions (phenylalanine, tyrosine, tryptophan, histidine, and cysteine).

This in silico analysis supports our experimental findings. Indeed, buckwheat albumin, the richest fraction in antioxidant amino acids, is the most active both in the intact and digested form. The redox amino acid content of buckwheat albumin is higher than the globulin fraction and this fits well with the observed difference in the DPPH scavenging activities of these fractions, especially in the digested forms. However, the experimentally determined high activity of digested amaranth HC globulin does not match with the calculated low content of antioxidant amino acids. Possibly, this could be due to the presence of co-purified polypeptides rich in antioxidant amino acids.

### 2.4. The Trypsin Inhibitor Activity of Protein Fractions and Peptides

The inhibitory activity was measured in vitro on the L-BAPA substrate by comparison with the activity of standard trypsin, set to 100%. Table 2 shows the inhibition percentages of the pseudocereal protein fractions. 

Buckwheat VLC and LC globulins were the most active, with about 79% and 83% of trypsin inhibition, respectively. The HC fraction showed no inhibitory activity. A remarkable inhibitory capacity was carried out also by albumin and VLC globulin of quinoa (74% and 65% inhibition, respectively). Among amaranth proteins, only LC and HC globulin fractions showed little inhibitory activity (about 50%); on the other hand, a low content of trypsin inhibitors (TI) has already been described for this species [69]. Different isoforms of buckwheat trypsin inhibitors with molecular weights between 5 to 7 KDa have been identified [70]. Subsequently, we evaluated the residual trypsin inhibitor activity of proteins after proteolysis. The results showed that TI activity is lower in all cases except, interestingly, in buckwheat albumin fraction, where it increased to up 100% after proteolysis, suggesting the possible release of strongly active peptides. To our knowledge, no previous report has been published describing the release of trypsin inhibitor activity by proteolysis of seed proteins. However, a peptide from a soybean Bowman–Birk inhibitor, containing 16 amino acid residues, with strong anti-tryptic activity has been described [71]. No activity was observed for all other protein hydrolyzed fractions tested. 

Serine proteinases have been demonstrated to be involved in immunity [72]: they can inhibit the proteases secreted at the time of injury or infection, which are the prime initiators of inflammation, and the release of neutrophils from lysosomes [73]. Our results indicate that the protein fractions with higher TI activity also possess radical scavenging and immune-modulating properties, supporting the hypothesis of the involvement of TI in protection against oxidation and inflammation. The Bowman–Birk protease inhibitor (BBI) from soybean is one of the most studied protease inhibitors. Different mechanisms were proposed to explain the BBI anti-proliferative and anti-inflammatory effects: the capacity to inhibit the proteolytic proteasome 20S activity [74], whose inhibition may result in the induction of apoptosis [75]; the inhibition of cathepsin G, elastase, and mast cell chymase, proteases involved in inflammatory processes [42]; and the reduction of the neutrophil infiltration and TNF-α during inflammation [76] and the increased production of IL-10, an anti-inflammatory cytokine that plays an important role in suppressing autoimmune diseases [34]. Intriguingly, seed protease inhibitors with antioxidant properties have been observed [73,77,78]. 

Overall, we analyzed, in vitro, the different biological effects of pseudocereal-derived proteins both in intact and in digested form. The evaluation of their bioavailabilities and metabolic fate was outside the scope of this work. However, previous studies have shown that some plant proteins introduced with the diet arrive in the intestinal tract in intact form and may be absorbed by intestinal cells [79]. The susceptibility of proteins and peptides to digestion and their bioavailability is influenced by diverse conditions, including extreme pH values and plasma membranes phospholipids interaction [80]. Considering this, the bioactivity of intact proteins may be improved by oral administration through gastro-protected tablets.

## 3. Materials and Methods

### 3.1. Protein Extraction and Purification

Sequential extraction of albumin and globulin protein classes was performed according to their differential solubilization. Seeds were ground to a meal until sifted through a 60-mesh sieve. Each of the following steps were performed twice. The flours were suspended in distilled water (1:10, *w*/*v*) and stirred for 4 h at 4 °C. The suspension was centrifuged at 10,000× *g* at 4 °C for 30 min to separate soluble albumin fraction. Pellets were resuspended (1:20, *w*/*v*) in a 50 mM sodium phosphate buffer, pH 7.5, containing 500 mM NaCl. The salt-soluble globulins were extracted for 4 h under stirring at 4 °C. The suspension was centrifuged at 10,000× *g* for 30 min at 4 °C and the supernatant was recovered. Albumins were dialyzed against water and freeze-dried. Globulins were gel-filtered using a Sephadex G-50 column, equilibrated in 50 mM phosphate buffer, pH 7.5, containing 100 mM NaCl. The desalted globulins were immediately loaded on a DEAE-cellulose column (20 × 180 mm) equilibrated with the same buffer (2 mg protein/mL resin). The unretained fractions were called VLC proteins, whereas the bound proteins were fractionated using the same buffer with stepwise addition of 150 and 250 mM NaCl (fractions LC and HC, respectively). All three fractions were then dialyzed, freeze-dried, and kept in sealed tubes at 4 °C until used.

### 3.2. SDS-PAGE

SDS-PAGE was carried out according to [81], on 12% polyacrylamide gel. For peptides separations, 16% polyacrylamide gels were used. Gels were stained by Coomassie Blue G-250 (BioRad, Milan, Italy.) Low-range SDS-PAGE Standards (Bio-Rad, Hercules, CA, USA) were used for *Mr* calculations

### 3.3. Protein Hydrolysis

Simulated gastro-intestinal digestion was carried out as previously described [50], focusing the action only on the proteolytic phase. Briefly, freeze-dried protein samples were dissolved at a concentration of 2 mg/mL in 0.01 M HCl. Pepsin solution was added to protein samples in a ratio 1:100 (*w*/*w*) and the samples were incubated at 37 °C for 10 min. The pH of the solution was then adjusted to 8.0 with 0.5 M NaOH and pancreatin was added to samples in a ratio 1:100 (*w*/*w*). The samples were incubated under shaking at 37 °C for 10 min. At the end, the reaction was stopped with a protease inhibitor cocktail (Merck Life Science, Milan, Italy) as recommended by the manufacturer.

### 3.4. Caco-2 Cells Cultivation and Immuno-Modulation Assay

All methods and protocols were essentially performed as previously described [45,82]. 

Cells were cultured in 75 cm^2^ flasks at 37 °C under 5% CO_2_, using DMEM supplemented with 10% inactivated fetal bovine serum, 2 mM L-glutamine, 100 U/mL penicillin, and 0.1 mg/mL streptomycin as growing medium.

Caco-2 cells were transfected with the plasmid pNiFty2-Luc (InvivoGen, Rho, Italy), after 24 h they were seeded in 24-multiwell plates at a density of 2 × 10^5^ cells/cm^2^. The immuno-modulation assay was then performed 24 h after transfection. The medium was replaced with fresh DMEM containing protein samples at the concentration of 1.0 mg/mL and incubated for 4 h at 37 °C. When appropriate, interleukin 1β (IL-1β) was added to the cell medium (10 ng/mL) for stimulating cell immune response. After incubation, plates were chilled on ice for 15 min and cells were scraped from the wells, transferred into 1.5 mL tubes and sonicated three times for 10 s on an ice bath, using a Soniprep 150 device (MSE, Heathfield, East Sussex, UK). After spinning, 100 μL of each supernatant was transferred in a 96-well microtitre plate (PerkinElmer, Waltham, MA, USA) and added with 25 μL of a solution containing ATP and D-luciferin (final concentrations: 1 mM and 10 μM, respectively. Luminescence was measured every 2 min using a Victor3 1420 Multilabel Counter (Perkin-Elmer, Waltham, MA, USA). The maximum recorded signal was considered for comparing the results.

For IL-8 expression quantifications, Caco-2 cells were seeded in 12-multiwell plates using complete DMEM and incubated as described until they reached confluence. Subsequently, cells were treated with 1 mg/mL of proteins or peptide samples for 1 h in the presence or absence of IL-1β (20 ng/mL), in complete DMEM. Total RNA was purified using the Aurum Total RNA Tissue Kit (Bio-Rad, Hercules, CA, USA) according to the manufacturer’s instructions. One μg total RNA was reverse transcribed into cDNA (20 μL final volume) using iScript Reverse Transcription Supermix for RT-qPCR kit (Bio-Rad, Hercules, CA, USA) and a Mastercycler Personal 5332 Thermal Cycler (Eppendorf Italia, Milano, Italy ). Reaction conditions were: 5 min at 25 °C, 20 min at 46 °C, and 1 min at 95 °C. The cDNAs were then diluted 1:100 with sterile water and 2 µl used as templates in quantitative PCR, using SsoAdvanced Universal SYBR Green Supermix and a CFX Connect Real-Time PCR detection system (Bio-Rad, Hercules, CA, USA). IL-8 specific primers were: 5′-ATGACTTCCAAGCTGGCCGTGGCT-3′ and 5′-TCTCAGCCCTCTTCAAAAACTTCTC-3′ [83]. The GAPDH reference gene was amplified with the following primers: 5′-GGAAGGTGAAGGTCGGAGTC-3′ and 5′-CACAAGCTTCCCGTTCTCAG-3′ [84]. Cycling conditions were: 3 min at 95 °C, then 40 cycles of denaturation (20 s at 95 °C), annealing (30 s at 55 °C), and extension (30 s at 72 °C). For each experiment, cDNA from unstimulated Caco-2 cells was used as the calibrator. Negative controls were performed without cDNA. Relative amounts of target genes compared to the GAPDH reference gene were calculated according to Livak [85]. Effects of the different molecules on inflammation were expressed as fold changes in target genes expression relative to the untreated control sample. Each individual treatment was performed in triplicate.

### 3.5. Antioxidant Activity Determinations

Antioxidant activities were assessed using the 2,2-diphenyl-1-picrylhydrazyl (DPPH) radical scavenging method, as previously described [86]. Briefly, 0.5 mL of DPPH (Merck Life Science, Milan, Italy) solution in methanol (0.03 mg/mL) were added to 0.5 mL of sample solutions at different concentrations and incubated for 15 min in the dark. Subsequently, the absorbance at 515 nm was measured by a spectrophotometer (Lambda 2, Perkin-Elmer, Waltham, MA, USA). The results were expressed as IC50, namely, the extract concentration that scavenged 50% of DPPH [87].

### 3.6. Trypsin Inhibitor Activity Assay

Trypsin inhibitor activity (TIA) was measured according to ISO 14902 standard method with slight modifications [88]. Trypsin activity was quantitatively determined by using the synthetic substrate Nα-Benzoyl-L-arginine 4-nitroanilide hydrochloride (BAPA, Merck Life Science, Milan, Italy). Trypsin stock solution was prepared dissolving 27 mg of trypsin (Merck Life Science, Milan, Italy) in 100 mL of 1 mM HCl with 5 mM CaCl_2_. This solution was then diluted 1:20 prior to the test execution. BAPA working solution was obtained diluting 1:100 of the stock solution (1.5 mM in DMSO) in 50 mM Tris-HCl, pH 8.2, and 5 mM CaCl_2_. The assay was performed by mixing 0.1 mL of protein extract (0,4 mg/mL) with 0.1 mL of BAPA working solution and 0.2 mL of water. After 10 min of incubation at 37 °C, 0.1 mL of trypsin solution was added and the sample was incubated for 10 min at 37 °C. The reaction was stopped by adding 0.1 mL of 30% acetic acid. The absorbance for the sample reading at 410 nm was a measure of the trypsin activity in the presence of the sample inhibitors (As). The reaction was also run in the absence of inhibitors by replacing the sample extract with an equal amount of water (standard). The corresponding absorbance was the reference reading (Ar). In addition, reagent blanks for the sample readings (Abs) and a reagent blank for the reference readings (Abr) were also prepared by adding the acetic acid solution before the trypsin solution. 

TIA was calculated as $i=\left[\frac{\left(\mathrm{Ar}\;-\;\mathrm{Abr}\right)\;-\;\left(\mathrm{As}\;-\;\mathrm{Abs}\right)}{\mathrm{Ar}\;-\;\mathrm{Abr}}\right]\;\times \;100$

Each experiment was performed in triplicate.

### 3.7. Statistical Analysis

Data reported in the histograms are expressed as the mean ± SE. Data from RT-*q*PCR were analyzed by using the CFX Maestro software (Bio-Rad, Hercules, CA, USA). Data were analyzed by a *t*-test; *p* values < 0.05 were considered statistically significant.

## 4. Conclusions

In this work, we explored three different biological effects exerted by each class of proteins and peptides derived from different pseudocereals, using biochemical methodologies and in vitro model cell systems. We designed our experiments so that the results complement what was previously reported. We also describe a fairly realistic depiction of what the gastro-intestinal proteolysis might produce. 

On the whole, the obtained results, thanks to the separations in single protein classes, alongside increasing knowledge about their individual effect, open new perspectives for their possible application, for example, in nutraceutical formulations.

## Figures and Tables

**Table 1:** Content of amino acids (AA) whose side chains may be involved in redox reactions.

	Quinoa		Amaranth		Buckwheat	
AA	Alb ^a^	Glob ^b^	Alb ^c^	Glob ^c^	Alb ^d^	Glob ^e^
**C**	6.1 ± 0.6	1.1 ± 0.3	1.9	1.5	7.9	0.9 ± 0.1
**F+Y**	2.9 ± 0.9	6.1 ± 0.9	5.9	4.8	4	5.9 ± 0.2
**W**	0.7 ± 0.1	0.8 ± 0.1	/	/	0.8	1.0 ± 0.3
**H**	1.4 ± 1.1	3.0 ± 0.4	2.3	2.3	0.8	2.3 ± 1.0
**Tot**	11.1 ± 1.5	11.0 ± 0.9	10.1	8.6	13.5	10.1 ± 1.1
***n***	2	4	/	/	1	3

Data are expressed as a percentage of the total amino acid number of the proteins (mean ± SD, where applicable). *n* = available sequence number (NCBI Proteins and UniprotKB databases accessed on 10 January 2020). ^a^ NCBI: XP_021758596.1, XP_021758543.1; ^b^ Capraro et al. [45]; ^c^ Segura-Nieto et al. [48]; ^d^ UniProt: Q2PS07; ^e^ UniProt: O23878, O23880, Q9XFM4.

**Table 2:** Trypsin inhibition percentage of protein fractions as such (native) and following simulated gastro-intestinal digestion (hydrolyzed).

	Fraction	Native	Hydrolyzed
Quinoa	Albumins	74.6 ± 6.2	2.1 ± 0.3
	VLC	65.5 ± 4.1	41.4 ± 2.8
	LC	51.8 ± 4.3	32.3 ± 3.2
	HC	42.3 ± 3.6	40.8 ± 2.4
Amaranth	Albumins	n.d. ^a^	46.3 ± 5.1
	VLC	41.5 ± 2.9	33.2 ±3.2
	LC	50.2 ± 5.7	31.9 ± 2.7
	HC	52.7 ± 3.5	21.7 ± 1.6
Buckwheat	Albumins	51.7 ± 6.8	101.5 ± 7.7
	VLC	79.6 ± 5.1	18.6 ± 1.8
	LC	83.6 ± 6.2	6.1 ± 3.9
	HC	0.4 ± 0.1	1.5 ± 0.2

^a^ n.d.: not determined.

## Data Availability

The data that support the findings of this study are available from the corresponding author upon reasonable request.
